# Plasma HGF concentration in patients with brain tumors

**DOI:** 10.3934/Neuroscience.2020008

**Published:** 2020-04-30

**Authors:** Zygmunt Siedlecki, Sebastian Grzyb, Danuta Rość, Maciej Śniegocki

**Affiliations:** 1 Department of Neurosurgery and Neurotraumatology and Pediatric Neurosurgery, Collegium Medicum in Bydgoszcz of Nicolaus Copernicus University in Toruń, Poland; 2 Department of Pathophysiology, Collegium Medicum in Bydgoszcz of Nicolaus Copernicus University in Toruń, Poland

**Keywords:** brain tumor, angiogenic factor, mitogenic factor, HGF

## Abstract

The Hepatocyte Growth Factor is a strong mitogenic factor and seems to play important role in tumor angiogenesis. The purpose of this study was to analyse the plasma concentration of this factor in patients treated surgically because of intracranial tumors. The study included 47 patients, both sexes treated surgically for intracranial tumors and 30 adult volunteers of both sexes, without cancer diagnosis. In study group 4 measurements of plasma HGF were taken: measurement 1: within 24 hours to 1 hour before the operation (preoperative), measurement 2: on the first day after the operation, i.e. after 24 hours, measurement 3: between the third and fifth day following the treatment, i.e. within 72–120 hours, and measurement 4: on the seventh day after the operation, i.e. after 840 hours. In control group only one measurement was taken. The distribution of the analyzed parameters was different from the normal distribution, therefore nonparametric statistics were used. The result values are presented in the form of a median (Me). The analysis revealed that HGR plasma levels in the patients with intracranial tumors in all 4 measurements (Me1 = 543.16 pg/ml, Me2 = 762.59 pg/ml, Me3 = 819.82 pg/ml, Me4 = 804.82 pg/ml) in the perioperative period were elevated in comparison to healthy subjects (Me = 361.04 pg/ml). The association has been shown to exist between postoperative HGF plasma levels and the clinical condition of patients with intracranial tumors (p = 0.0342). Postoperative HGF levels correlated negatively with the patients' postoperative condition. It was also found that in patients with supratentorial tumors HGF plasma levels were higher (Me = 557.74 pg/ml) in comparison to patients with posterior fossa tumors (Me = 325.00 pg/ml). These results suggest increased angiogenic and mitogenic activity in patients with intracranial tumors and its even greater intensity in the postoperative period. Greater angiogenic activity appears to occur in patients with supratentorial tumors.

## 1. Introduction

The Hepatocyte Growth Factor (HGF) is a protein that exhibits strong mitogenic properties—one that regulates the development and regeneration of tissues [1]. It is produced by all cells of mesenchymal origin. Characterized by the ability to disperse colonies of endothelial cells and hepatocytes, it is also called the “scatter factor” (SF). HGF is a heterodimeric protein composed of 728 amino acids and consisting of the heavy chain with a weight of weight 69 kDa and a light chain weighing 34 kDa, bound together by a disulphide bond [1],[2]. HGF binds to its membrane c-Met receptor with tyrosine kinase activity. The protein of the c-Met receptor is encoded by a proto-oncogene with the same name, whose overexpression has been demonstrated in many cancer diseases. Binding of HGF to c-Met activates metabolic pathways, resulting in an increase in the synthesis of metalloproteinase-1 and stromyelisine-1, responsible for the degradation of the basement membrane of the endothelial cells and the extracellular matrix. HGF shows angiogenic properties, as it stimulates degradation of the endothelial basement membrane, but also due to activating tumor cells to secrete other growth factors. HGF inhibits apoptosis of tumor cells. It was also shown that it prevents deoxyribonucleic acid (DNA) damage in breast cancer cells and in high-grade gliomas. By stimulating cell proliferation and angiogenesis, HGF promotes tumor metastasis. Elevated levels of HGF have been shown in patients with metastatic breast cancer. Numerous reports in the literature demonstrate great importance of HGF in the pathogenesis of cancer, including intracranial tumors [1],[2]. HGF has been proven important in stimulating the proliferation of new blood vessels in glial tumors. As a powerful mitogen, HGF stimulates mitotic division in glial cells, endothelial cells and in the tumor cells themselves, by binding to its MET receptor [1],[3]. Increased expression of HGF and MET protein correlates with the degree of vascularization and with the malignancy of glial tumors [1],[2]. HGF binding to c-Met leads to the initiation of series of cascade mediating wound healing and embryogenesis. However, in cancer cells, mutation in c-Met stimulates pathways causing aberrant c-Met/HGF axis activation and resulting in development and progression through migration, invasion, and metabolic reprogramming in cancer [4]. HGF is a useful biomarker to predict gastric cancer risk and may play potential role in primary prevention of gastric cancer [5]. Preoperative serum HGF is a tumor peripheral biomarker of breast cancer that could predict the prognosis of breast cancer [6]. It was proven that in patients with more advanced breast and colorectal cancers and the presence of metastases, HGF levels were significantly higher than in patients with less advanced proliferative process [6],[7].

## 2. Materials and methods

In order to conduct the research, the approval of the Bioethics Committee of Nicolaus Copernicus University Collegium Medicum in Bydgoszcz was obtained, its number being: KB-665/2009. The test groups were determined as follows: b—study group, p—comparative group. The study included 47 adult patients (Mb = 57.52) of both sexes (22 women, 25 men) treated surgically for intracranial tumors in the Department of Neurosurgery and Neurotraumatology at University Hospital No. 1, Ludwik Rydygier Medical School in Bydgoszcz of Nicolaus Copernicus University in Toruń. The diagnosis of intracranial tumors was based on medical history, physical examination, neuroimaging, and, in the postoperative period, on the basis of histopathological examination. As some of the patients had been transferred from other surgery centers in Poland, they had already been partially diagnosed at the time of admission, including neuroimaging tests. Although patients with solitary intracranial metastatic tumors were in the study group, the study excluded patients with disseminated metastatic disease with multiple metastases and patients who underwent surgery within less than 30 days prior to admission to the Neurosurgery and Neurotraumatology Clinic. Also excluded were patients with pre-existing consciousness disorders due to intracranial tightness, which prevented them from giving informed consent. In addition, the study group also excluded patients who, due to acute complications, required a subsequent operation during the study period, i.e. within seven days following the surgery.

The control group consisted of 30 adults (Mp = 58.16) volunteers of both sexes (14 women and 16 men), without tumor diagnosis, who did not underwent surgery in less than 30 days, negating occurrence of diabetes, hypertension and coronary heart disease, and do not taking any medicines on a regular basis. The control group consisted of persons completely independent of the author, they were not in any official dependencies, they were not in any private dependencies. All the people who were qualified for the study, after being informed of its subject, gave their written consent to taking part in it. Study group and control group did not show statistically significant differences in sex (p = 0.71) and age (Mb = 57.52; Mp = 58.16; t = 0.4355; p = 0.69).

In study group among the examined patients with tumors of various degrees of histological malignancy, 34.04% neoplasms were grade II according to the 4-grade WHO scale. Other tumors in the range of malignancy occurred in the study group with frequency 29.79% for III WHO, 21.28% for IV WHO and 14.89% for I WHO. The frequency of the occurrence of the particular tumor types confirmed by histopathological finding was assessed by the division into the 4 groups: gliomas (38.33%), metastases of cancer (23.40%), meningiomas (23.40%) and intracranial tumors identified as other (14.89%). The last group were vestibular schwannomas and pituitary adenomas. Glial tumors diagnosed in 38.29% of cases were of various degree of malignancy according to WHO (II-IV).

In study group 78.72% tumors were located supratentorial and 14.89% tumors in the posterior cranial cavity—infratentorial, including tumors of cerebellum and cerebellopontine angle. In 6.38% cases the tumors were located in sellar and parasellar region and were classified in this manuscipt as other location.

The functional and clinical status of patients was assessed on the basis of the Karofofsky scale (0–100). The postoperative functional state that presented in this manuscript was good (80–100) in 80.85% of patients, medium (50–70) in 8.51% and poor (20–40) in 10.64% of patients.

In the study 4 blood samples were taken. The first sample was taken within 24 hours to 1 hour before the operation (measurement 1), the second sample was collected on the first day after the operation, i.e. after 24 hours (measurement 2), the third sample was taken between the third and fifth day after treatment, i.e. within 72–120 hours (measurement 3), while the fourth sample was taken on the seventh day after the operation, i.e. after 840 hours (measurement 4). As the operations took place in the morning, the measurements were also taken in the morning, when the patients were on an empty stomach. Venous blood was collected from direct punctures of the basilic vein, median cubital vein or median antebrachial vein, following skin disinfection with the antibacterial agent Kodan® Tinktur Forte, using the Vacuette® vacuum blood collection system from Greiner Bio-One. Venous blood was collected into two plastic tubes with sodium citrate at a concentration of 3.2% edetic acid (EDTA) in a ratio of 1:10—one part of anticoagulant to nine parts of blood in the tube. The samples were collected in accordance with the current blood collection procedure used at the Department of Neurosurgery and Neurotraumatology of Nicolaus Copernicus University Medical School. In order to obtain platelet-poor plasma, the blood samples were centrifuged in a refrigerated centrifuge at 4 °C for 20 minutes at the speed of 3000 rpm. The obtained citrate plasma and EDTA plasma was separated into portions of approximately 200 µl in Eppendorf tubes. The material was frozen at −80 °C until the time of testing. Concentrations of individual factors were determined using reagent kits from R&D and Bender MedSystems. In the plasma, the level of hepatocyte growth factor (HGF) was marked using R&D Human HGF reagent; the reference values were 251–742 pg/dl. The study used the ELISA immunoassay method. The tests were carried out in the Hemostatic Disorders Laboratory of the Department of Pathophysiology, Nicolaus Copernicus University's Medical School in Bydgoszcz.

For determining the parameters under investigation, in all cases, the study only used samples of patient blood collected in order to carry out laboratory tests required for diagnostics and therapy during hospitalization. All patients participating in the study had been informed of the nature and purpose of the study and gave informed consent for further measurements of plasma HGF levels. In the control group, a single venous blood sample was taken and plasma concentrations of the required factors were measured. The control group consisted of healthy volunteers—acquaintances of the author, who were not in any business relationship with him. No cases of cancer were reported in the medical histories of the control group. In addition, no control group members had been treated surgically within 30 days prior to obtaining the samples. None of them suffered from hypertension, ischemic heart disease or diabetes, and they were not taking any medications on a permanent basis at the time.

### 2.1. Statistical analysis

The statistical analysis was carried out using StatSoft® statistical program STATISTICA 8.0. The distribution of the analyzed parameters was different from the normal distribution, therefore nonparametric statistics were used: Kruskal-Wallis and Friedman test.

The significance of differences between two groups was assessed using the Mann-Whitney U test for independent variables and Wilcoxon for dependent variables. The significance level of p < 0.05 was assumed to be statistically significant.

The Spearman correlation coefficients used in the correlation analysis were also considered statistically significant for p < 0.05. The interdependence of the analyzed parameters is presented graphically in the form of scatterplots with linear regression curves.

## 3. Results

**Figure 1.**
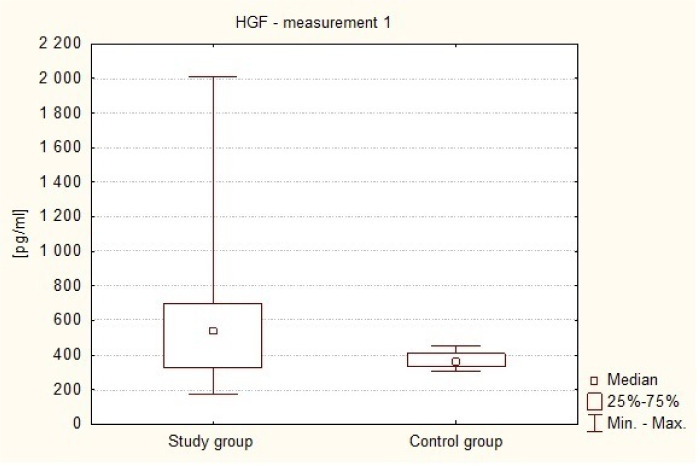
Preoperative plasma levels of HGF in patients and healthy volunteers.

It was found that preoperative plasma levels of the HGF in brain tumor patients were higher (Me = 543.16 pg/ml) than the levels found in healthy subjects (Me = 361.04 pg/ml). This difference was statistically significant (p = 0.0036 in the Mann-Whitney U test). This is illustrated in Figure 1.

**Figure 2.**
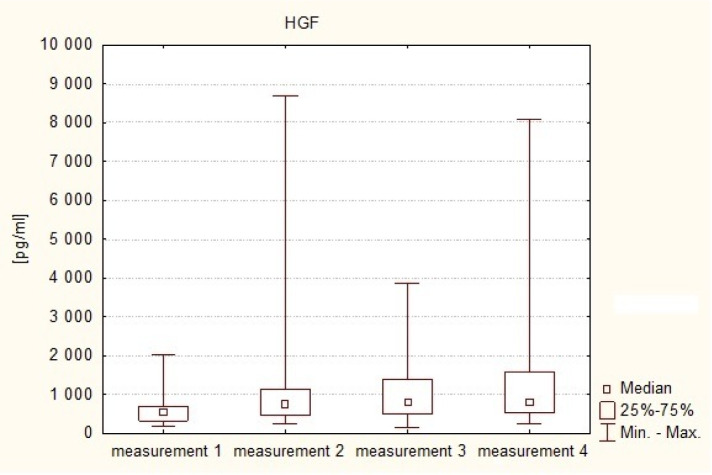
HGF serum levels in consecutive measurements in the study group.

The plasma HGF levels on the first day after the operation (measurement 2) were higher (Me = 762.59 pg/ml) than in the preoperative measurement (Me = 543.16 pg/ml). On the 3rd–5 postoperative day (measurement 3) a further increase in the levels of this factor was shown (Me = 819.82 pg/ml), while in the fourth assay (measurement 4) a decrease in HGF was observed (Me = 804.82 pg/ml), although its value was still higher than on the first day after the operation, and markedly higher than in the preoperative measurement. Statistically significant differences were shown to exist between the preoperative measurement of HGF level and each of the three postoperative measurements (Friedman test: measurement 1 vs. measurement 2—p = 0.001, measurement 1 vs. measuring 3—p = 0.001, and measurement 1 vs. measurement 4—p = 0.001). This means that in patients with intracranial tumors in the postoperative period there is an increase in plasma levels of the angiogenic factor, i.e. HGF. This is illustrated in Figure 2.

It was also observed that, in all the measurements during the postoperative period, i.e. on the first day, between the third and fifth day, and on the seventh day after the treatment, serum concentrations of HGF were higher (Me2 = 762.59 pg/ml, Me3 = 819.82 pg/ml, Me4 = 804.82 pg/ml) than the values of this parameter in the control group (Me = 361.04 pg/ml).

**Figure 3.**
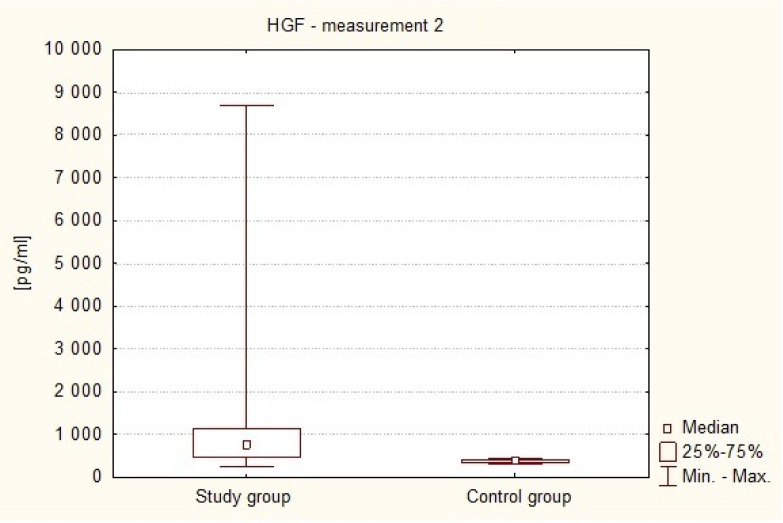
Plasma levels of HGF on the first day after surgery in the study group and in healthy volunteers.

Figure 3 shows the difference between serum HGF concentrations in patients with intracranial cancer on the first day after surgery (Me2 = 762.59 pg/ml) and in healthy volunteers (Me = 361.04 pg/ml). It was observed that the value of this parameter was significantly higher in the study subjects than in the control group (p < 0.0001 in Mann-Whitney U test).

**Figure 4.**
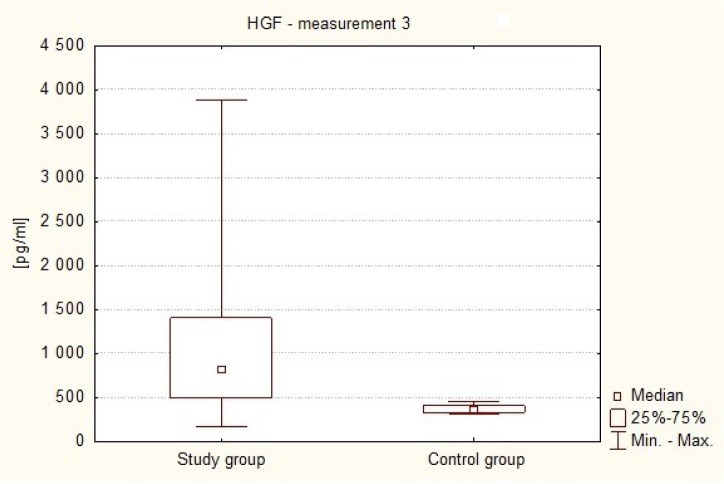
HGF plasma levels on the 3rd–5th day after the surgery in the study subjects and in healthy volunteers.

Figure 4 shows the difference between the concentrations of serum HGF in patients with brain tumors in the period between the third and fifth day after the surgery (Me3 = 819.82 pg/ml) and in healthy volunteers (Me = 361.04 pg/ml). It was observed that the value of this parameter was significantly higher in the study subjects than in the control group (p < 0.0001 in the Mann-Whitney U test).

**Figure 5.**
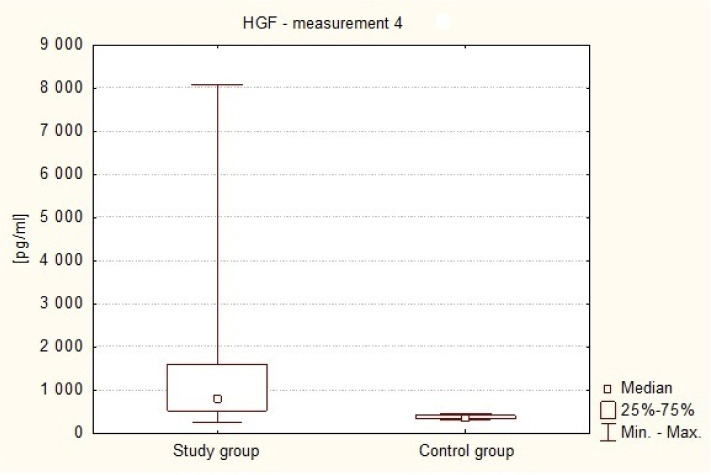
HGF plasma levels on the 7th day after surgery in the subjects and in healthy volunteers.

**Figure 6.**
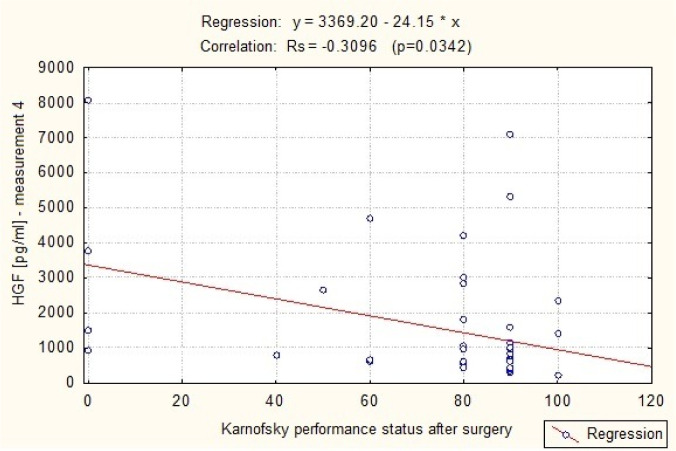
A scatterplot of the HGF regression curve (measurement 4) and Karnofsky performance scale on the seventh day after surgery.

Figure 5 shows the difference in serum HGF levels between patients on the seventh day after the operation (Me4 = 804.82 pg/ml) and in healthy volunteers (Me = 361.04 pg/ml). HGF plasma levels were significantly higher in the patients compared with the control group (P < 0.0001 in the Mann-Whitney U test). A correlation was demonstrated to exist between plasma levels of the angiogenic factor HGF on the seventh day following the surgery (measurement 4) and the funcjonal condition of the patients at the time, using the Karnofsky performance score. A statistically significant negative correlation (p = 0.0342) between plasma HGF concentration and the number of points on the scale. This means that the subjects with lower HGF levels in the blood on the seventh day after the operation of intracranial tumor were in significantly better clinical condition. This is shown in Table 1 and in Figure 6.

**Table 1.** The values of Spearman's rank correlation coefficient between plasma levels of the measured parameters and the clinical status of the study subjects after the surgery.

Parameter	Clinical condition after the surgery	p
HGF	−0.3096	0.0342

**Figure 7.**
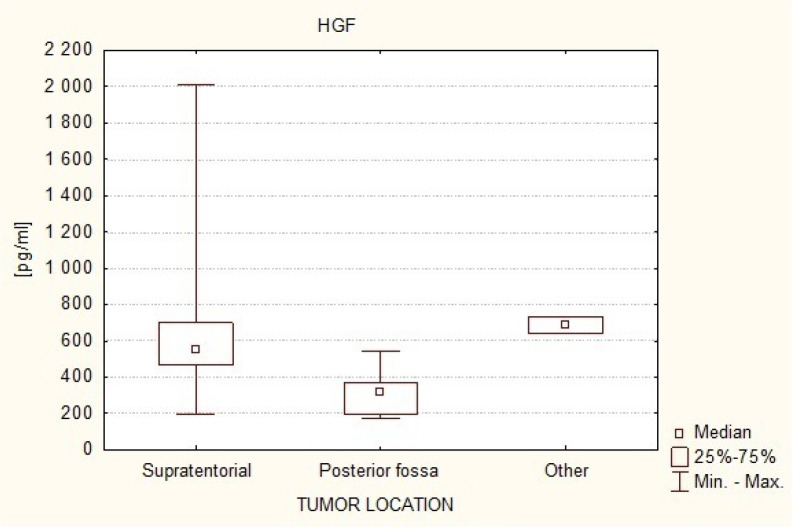
Serum HGF levels in the study subjects depending on the location of the intracranial tumor.

Figure 7 illustrates the differences in HGF plasma levels (measurement 1) between patients with supratentorial tumors (Me = 557.74 pg/ml) and patients with tumors of the posterior fossa (Me = 325.00 pg/ml) and tumors in other locations (Me = 697.84 pg/ml). It was observed that HGF plasma levels are significantly lower in patients with infratentorial tumors compared to supratentorial tumors and to tumors in different locations (Kruskal-Wallis test: 1 vs. 2 p = 0.0167; 2 vs. 3 p = 0.0253).

Analyzing HGF plasma level depending on the grade of malignancy according to WHO, no statistically significant differences were found between the values of HGF plasma level in all measurements (1–4) and the grade of tumor malignancy (I–IV). Neither no statistically significant was found between plasma HGF level in all measurements (1–4) and the histopathological type of intracranial tumors.

## 4. Discussion

This research also showed significantly elevated levels of HGF in the patients with brain tumors, both before and after the surgery, compared to the control group of healthy volunteers. HGF is a multifunctional growth factor that stimulates the proliferation, morphogenesis, and survival of cancer cells [1],[8], including cancers of the brain [9]. Laszmus et al. in 1999 conducted a study which found that in certain types of cancer such as glial brain tumors and breast cancer HGF stimulates tumor cell survival and invasiveness, and it increases protease activity and directly stimulates division of endothelial cells, playing a role in the creation of new blood vessels [9]. The Laszmus studies relied on measuring secretion of HGF proteins in glioma cells using the ELISA method. Twenty glioma samples were examined initially, from which protein extracts were obtained, and elevated HGF were found in the tumor cells. This test was supplemented by further 73 measurements of glioma samples and it was demonstrated that HGF levels in high-grade glial tumor tissue is significantly higher than in low-grade tumors, and that HGF levels in glioma cells are significantly higher than HGF levels in brain tissue free from neoplasm, in patients treated surgically for epilepsy [9]. Significantly increased HGF expression was also found in the endothelial cells of blood vessels in high-grade gliomas and in glial astrocytes found in the invasive glioma growth [9]. In the present paper, HGF levels were also measured using the ELISA method, but in contrast to the studies made by Laszmus et al., the measurements were made in the plasma collected from a peripheral vein, rather than the tumor tissue, the brain, or vascular endothelial cells. The present study also included patients with various intracranial tumors, among which gliomas constituted 38.3% of the group. However, significantly higher HGF levels in the blood of the patients with intracranial tumors compared with healthy subjects appear to be consistent with the conclusions of the studies of Laszmus et al., proving the importance HGF in the development of cancer. Also consistent with the research of Laszmus et al. are the results of Koochekpour's work (1997), in which he found that the level of HGF expression within the gliomas is positively correlated with their degree of malignancy [10]. Rao et al. [11] in his study of 1997 found that in tumors originating from the nerve sheath, HGF is expressed in high-grade tumors, and it is not found in low-grade tumors. This demonstrates that HGF expression in intracranial tumors may be an indicator for distinguishing low and high grade tumors [11]. This finding is not confirmed by the results of my work, as I have not found any significant differences in plasma concentrations of HGF in relation to the degree of malignancy of the tumor and its histological type. In contrast to the studies of Koochekpour [10] and Rao [11], the present study examined plasma levels of HGF rather than its expression in the tumor tissue. In examining the importance of HGF concentrations in the blood of cancer patients, we may refer to the work Maemura et al from 1998 [12], which measured the concentration of HGF in the serum of 34 patients with breast cancer in metastatic stage. It was shown that the concentration of HGF in the serum was significantly higher in patients with more advanced stages of metastatic cancer than in patients with less advanced cancer. It was also found that patients with higher levels of HGF in the serum showed shorter survival times than patients with lower levels of the factor, which indicates that the concentration of HGF in the serum may be a useful indicator of the stage of neoplastic disease and the related outcomes. Pai in his study in 2020 also showed that preoperative HGF levels are higher in patients with more advanced breast cancer with metastases compared to patients with less advanced cancer [6]. This is consistent with the results of this study where preoperative HGF plasma level in patients with brain tumors was also higher. In my studies I have shown that higher postoperative plasma concentrations of HGF were observed in individuals in worse functional clinical condition, whereas in patients in better condition HGF levels were lower. Although my results cannot be directly related to Maemura's results [12], considering the fact that the clinical status of patients with intracranial tumors is related to prognosis, also elevated HGF levels in the blood of patients with intracranial tumors seem to be associated with a worse course of illness and prognosis. The prognostic value of HGF was also indicated by Moriyama in his study published in 1997 [13]. He demonstrated that increased expression of HGF may be associated with a worse prognosis in patients with high-grade gliomas. In the present study I have also shown that plasma HGF levels increase after the operation with the highest concentration between the third and fifth day after the operation. All postoperative HGF levels were significantly higher than in the preoperative tests. In the literature, there are no similar reports on changes in HGF concentrations after neurosurgical operations. Kurumiya [14] described the increased production of HGF into the bile ducts after operations of the liver and bile ducts, but these results cannot be related to the results of my work, as they do not speak so much of increased expression of HGF after operations in general, but only of the role of bile ducts in the production of this growth factor [15]. HGF, as a highly mitogenic substance, is involved in the regeneration of tissues, including the healing of wounds. Nayeri et al. [16] showed in 2006 that this particular growth factor is produced in the healing process in the wake of damage resulting from skin burns [17],[18]. My research has also shown that plasma HGF levels are significantly lower in patients with tumors in the posterior cranial fossa, compared to patients with supratentorial tumors and tumors in other locations. In the available literature there are no similar reports. Maemura's paper [12] on the concentration of HGF in the serum of patients with metastatic breast cancer showed that it has no relationship to the location of the metastatic focus [8],[19],[20]. The mitogenic activity of HGF is important in wound healing and tissue regeneration and renewal processes. Reports on this subject are described in the literature on both human and animal models. Komaki et al. in 2019 proved the importance of HGF in the rat tissue repair and ulcer healing process. In his study administration of HGF for 7 days led to a significant reduction in the ulcerative area and enhanced the proliferation of esophageal epithelial cells. HGF treatment significantly decreased the fibrosis. HGF facilitates the repair of esophageal mucosal injury and may also ameliorate the esophageal fibrosis, possibly through enhanced re-epithelization [21]. Wang and et obtained similar results in their manuscript in 2017 documenting a positive effect of HGF on ulcer healing by reducing inflammation [22]. According Miyagi et al. (2018) the role of HGF plays important role in corneal stromal and endothelial wound healing and it is even understudied. In addition, HGF has been shown to play an anti-fibrotic role by inhibiting myofibroblast generation and subsequent production of a disorganized extracellular matrix and tissue fibrosis [23]. Therefore, HGF represents a potential therapeutic tool in numerous organs in which myofibroblasts are responsible for tissue scarring [21]–[23]. According to Chen et al. (2019) HFG plays a role in physiological processes in the area of white adipose tissue (WAT) [24]. Mamy studies on mice have attested the roles of dermal white adipose tissue in wound healing [24],[25]. The importance of HGF in wound healing processes described in the literature seems to be consistent with the results obtained in this manuscript. In the studied group, high HGF values were obtained in patients from the 7th day after surgery, i.e. when tissue healing processes are active.

## 5. Conclusions

Based on the obtained results and statistical analyzes, the following conclusions were drawn:

1. In patients with intracranial tumors in the perioperative period there are increased plasma levels of the proangiogenic factor, i.e. HGF, compared to people with no cancer diagnosis, as evidenced by increased angiogenic and mitogenic activity in these patients.

2. In the postoperative period there is an increase of HGF plasma levels. This may indicate an increase in angiogenic and mitogenic activity in the first week following surgical treatment of intracranial tumors. Based on the literature, high mitotic activity may result from the wound healing process and seems not be due to neoplasm disease. Especially since patients with disseminated metastatic process were excluded from the study.

3. The concentration of HGF is associated with the perioperative clinical and functional status of patients with intracranial tumors: postoperative HGF levels correlate negatively with the patients' condition.

4. Plasma HGF levels are associated with intracranial tumor location. Higher levels of HGF are present in supratentorial tumors than infratentorial tumors.
